# Longitudinal Changes of Retinal Structure in Molecularly Confirmed *C1QTNF5* Patients With Late-Onset Retinal Degeneration

**DOI:** 10.1167/tvst.12.12.14

**Published:** 2023-12-12

**Authors:** Riccardo Cheloni, Ashwin Venkatesh, Ana Catalina Rodriguez-Martinez, Mariya Moosajee

**Affiliations:** 1 UCL Institute of Ophthalmology, London, UK; 2 Moorfields Eye Hospital NHS Foundation Trust, London, UK; 3 The Francis Crick Institute, London, UK

**Keywords:** *C1QTNF5* gene, natural history, late-onset retinal degeneration (LORD), fundus autofluorescence (FAF)

## Abstract

**Purpose:**

The purpose of this study was to present our findings on the natural history of late-onset retinal degeneration (LORD) in patients with molecularly confirmed *C1QTNF5* heterozygous pathogenic variants and assess suitability of retinal structure parameters for disease monitoring.

**Methods:**

Sixteen patients with *C1QTNF5-*LORD were retrospectively identified from Moorfields Eye Hospital, UK. Fundus autofluorescence (FAF), optical coherence tomography (OCT) scans, and best-corrected visual acuity (BCVA) were collected. Area of atrophy (AA) was manually drawn in FAF images. Ellipsoid zone (EZ) width and foveal retinal thickness of the whole retina and outer retina were extracted from OCT scans. Age-related changes were tested with linear-mixed models.

**Results:**

Patients had median age of 62.3 years (interquartile range [IQR] = 58.8–65.4 years) at baseline, and median follow-up of 5.1 years (IQR = 2.6–7.6 years). AA, EZ width, and retinal thickness parameters remained unchanged until age 50 years, but showed significant change with age thereafter (all *P* < 0.0001). AA and EZ width progressed rapidly (dynamic range normalized rates = 4.3–4.5%/year) from age 53.9 and 50.8 years (estimated inflection points), respectively. Retinal thickness parameters showed slower progression rates (range = 1.6–2.5%/year) from age 60 to 62.3. BCVA (median = 0.3 LogMAR, IQR = 0.0–1.0 at baseline) showed a rapid decline (3.3%) from age 70 years. Findings from patients with earlier disease showed FAF atrophy manifests in the temporal retina initially, and then progresses nasally.

**Conclusions:**

Patients with LORD remained asymptomatic until age 50 years, before suffering rapid outer retinal degeneration. EZ width and AA showed rapid progression and high interocular correlation, representing promising outcome metrics. Clinical measures also capturing the temporal retina may be preferable, enabling earlier detection and better disease monitoring.

**Translational Relevance:**

Area of atrophy in FAF images and OCT-measured EZ width represent promising outcome metrics for disease monitoring in patients with *C1QTNF5-*LORD.

## Introduction

Late-onset retinal degeneration (LORD) is an autosomal dominant inherited retinal disease, first recognized around 20 years ago.^1^^–^^3^ Its prevalence remains largely undetermined with over 100 molecularly confirmed cases reported in the literature at present, but many cases may be under-reported.^4^ It arises from heterozygous mutations involving the *C1QTNF5* gene (OMIM: 605670 and 608752).^1^ To date, 6 variants have been reported,^4^ and the founder missense mutation c.489C>G p.(Ser163Arg) was the first discovered and most prevalent.^1^ *C1QTNF5* has 3 exons (7764 bases) encoding a 243 amino acid protein, that is part of the adiponectin family.^5^ Although the precise function of C1QTNF5 is yet to be fully characterized, this protein is largely expressed in the ciliary body and the retinal pigment epithelium (RPE),^6^^,^^7^ and plays a role in cellular adhesion and in regulating cellular fatty acid metabolism.^8^ Recently, a study using LORD induced pluripotent stem cells showed sustained activation of the AMP-activated protein kinase (AMPK) metabolism, resulting in the loss of cellular sensitivity to changes in AMP/ATP ratio.^8^

Clinically, LORD presents as a macular degeneration, in early disease, and can be misdiagnosed as age-related macular degeneration (AMD). The disease primarily affects RPE, with subsequent changes of the outer retina and choroid, with a good degree of symmetry but some phenotypic variability.^9^ Night blindness is widely reported as the presenting symptom, between ages 45 and 55 years.^9^^,^^10^ Long anteriorly inserted zonules (long zonules) are a distinctive early sign of LORD and can often be found concurrently with peri-pupillary atrophy, when patients are asymptomatic and without detectable fundus changes.^6^^,^^11^

Three severity stages have been conventionally proposed in LORD^12^; (1) an asymptomatic phase without fundus changes until age 40 years with long zonules being the sole abnormality, (2) peri-macular yellow dots in the fundus with pigmentary changes in the midperiphery between ages 40 and 60 years, and (3) atrophic fundus changes and typical scalloped atrophy on fundus autofluorescence (FAF) from 60 years+.

More recent work has supplemented our understanding of the clinical course of LORD with spectral domain optical coherence tomography (SD-OCT) and FAF imaging.^9^^,^^13^^–^^17^ Early changes include subretinal modifications resembling reticular pseudo-drusen.^17^ These lesions present as focal hypo-autofluorescence in areas of increased autofluorescence with evidence of subretinal deposits on OCT scans in an otherwise preserved outer retina. Disease progression is marked by transition to speckled FAF, with OCT scans revealing patchy ellipsoid zone (EZ) loss, RPE-Bruch's membrane separation, and thinning of the outer retinal layers. Later LORD stages are characterized by diffuse atrophy on FAF, which originates in scalloped areas of hypo-autofluorescence and then becoming confluent. OCT scans at this stage present with severe outer retina atrophy and display hyper-transmission where the RPE has degenerated. Recent studies, including patients harboring variants other than the most common p.(Ser163Arg), have also highlighted phenotypic variability demanding further characterization of clinical presentation across LORD genotypes.^10^^,^^18^

Although a few therapeutic avenues are currently being explored for LORD,^6^^,^^8^^,^^19^ the disease remains untreatable, and patients progress to severe visual impairment by age 70 years.^4^ The development of novel treatments through rigorous clinical trial scrutiny requires a thorough understanding of disease progression, with knowledge of suitable outcome metrics and appropriate selection of study participants. Despite key clinical signs and progression patterns being described, longitudinal studies assessing quantitative measures of LORD disease progression are lacking. In this study, we characterized the clinical course of patients with a molecularly confirmed *C1QTNF5-*LORD diagnosis, with a focus on the progression of retinal structure measures. Our findings will facilitate the development of outcome metrics for future clinical trials, and will contribute to better prognostication for patients with LORD.

## Methods

### Setting and Study Population

Patients with a confirmed molecular diagnosis of *C1QTNF5*-LORD were retrospectively identified from the Moorfields Eye Hospital Inherited Eye Disease Database (Moorfields Eye Hospital NHS Foundation Trust, London, UK). All study participants gave informed consent to participate, and all procedures were conducted in adherence to the tenets of the Declaration of Helsinki. The study received ethical approval from the relevant local bodies (Research Ethics Number: 12/LO/0141). Seven patients were included here which were first reported in previous observational series from our institution.^9^^,^^18^ Patient ID numbers were not reported in previous publications to facilitate cross-reference.

Data for this study were collected as part of standard clinical care and retrospectively analyzed. For all participants, a full eye examination was conducted at each visit, including best-corrected visual acuity (BCVA), and multi-modal retinal imaging. We extracted data from the electronic and written medical records of each patient.

A clinical diagnosis of LORD and molecular confirmation of disease-causing variants in *C1QTNF5* were the main inclusion criteria. Methodology of genetic testing and variant interpretation at Moorfields has been described previously.^20^^–^^22^ Patients with retinal changes due to disease other than LORD were excluded. Where eligible, both eyes of each patient were included.

Visual field and electrophysiology examinations were available in a minority of patients, with inconsistent testing protocol, and at a single time point. Accordingly, these were not considered in this longitudinal analysis and BCVA was the sole functional parameter assessed.

### Retinal Imaging

Macular SD-OCT scans and short wavelength FAF imaging were conducted with the Spectralis (Heidelberg Engineering, Heidelberg, Germany). Macular cube scans were performed with varying scan pattern: either 19 B-scans (512 A-scans/B-scans) or 97 B-scans (1024 A-scans/B-scans) centered on the fovea. The built-in automated retinal tracking was used to reduce measurement noise in OCT examinations, and follow-up OCT scans were registered to baseline. Wide-field pseudo-color fundus photographs were collected with Optos California (Optos plc, Dunfermline, UK). Fundus photographs at baseline were qualitatively graded by one experienced clinician (author R.C.) according to the staging proposed by Borooah et al.^12^; (1) no fundus changes, (2) peri-macular or peripheral yellow dots, and pigmentary changes in the midperiphery, and (3) atrophic fundus changes.

### Fundus Autofluorescence Images

FAF images were qualitatively graded by one experienced clinician (author R.C.), according to previously described modifications^14^: (1) normal autofluorescence, (2) reticular pseudo-drusen or speckled changes, and (3) atrophic changes. To quantify disease progression in FAF, we measured the area of atrophy (AA), which was assessed at all visits and from both eyes of each patient, where available. All image analysis was performed in MATLAB (version 9.6.0; The MathWorks Inc., Natick, MA, USA). FAF images from Spectralis (tiff format) were read into MATLAB, and the left eyes were flipped to the right eye format for consistency. By also using the OCT scan as guidance, the fovea and the optic disc were subjectively identified in baseline FAF images. The pixel lateral resolution was extracted from the Spectralis FAF examination, to provide a conversion factor from pixel to µm. Regions of atrophy were then manually segmented by one experienced clinician (author R.C.) according to written instruction and in a standardized setting. For this task, we used the manual drawing of region of interest on the ImageSegmenter app, available in MATLAB Image Processing Toolbox. According to previous criteria,^23^^,^^24^ atrophy was defined as homogeneous areas of well-demarcated definite decreased autofluorescence. Where FAF images were inconclusive, the presence of atrophy was further evaluated on SD-OCT scans and defined as a loss of retinal pigment epithelium with or without changes of the outer retinal. Areas of peripapillary atrophy, if present, were also segmented and excluded from analysis to avoid ambiguity. Last, atrophy segmentations were reviewed by a second experienced clinician (author A.C.R.M.).

In patients with multiple examinations, follow-up images underwent registration to baseline before any additional analysis to ensure consistency with follow-up. This was achieved via the MATLAB built-in control points selection tool (cpSelect function). Coordinates of corresponding points were extracted from the baseline image and the follow-up image. A minimum of 9 points representing the same retinal detail (e.g. blood-vessel intersection) were manually identified, and were then used to compute a geometric projective transformation that aligns follow-up image with that at baseline. After registration, follow-up images underwent the same analysis described above.

As a quantitative measure of disease progression, we measured AA within the 6 mm diameter circle of the Early Treatment Diabetic Retinopathy Study (ETDRS) grid, and the largest circle capturing most of the 55 degrees FAF field (14 mm diameter), both centered on the fovea. Atrophy was manually segmented in all available FAF images, but AA was only extracted from 55 degrees field images to also include the temporal retina that may often show atrophy earlier.^9^^,^^13^^,^^14^ Measurements of AA were square root transformed to reduce bias from baseline lesion size on growth rate estimates.^15^^,^^25^

**Figure 1.**
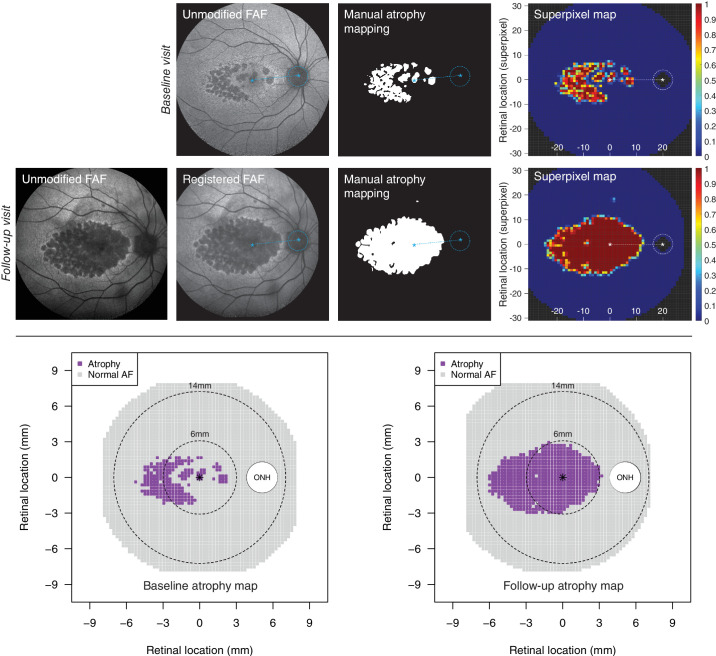
Example of analysis of atrophy in fundus autofluorescence (FAF) images. The *top panel* shows baseline and follow-up FAF images. The outcome from the manual segmentation of atrophy is reported, and this image underwent shear transformation to bring the fovea and the optic nerve head (ONH) on a common horizontal axis. In the superpixel map, the fovea and ONH are aligned and located at a consistent position across all eyes (fovea = [0; 0]; ONH = [20; 0]). Both x and y axes report retinal location in superpixel and each superpixel is color-coded according to the proportion of atrophic pixel within the superpixel. At follow-up, the FAF image was registered to baseline and the same processing was applied. The *bottom panel* shows the atrophy maps at baseline and follow-up, also including the 6 mm and 14 mm diameter circles considered to quantify area of atrophy (AA). Each superpixel was considered abnormal when at least 50% of included pixels were atrophic (i.e. ≥ 0.5).

In order to establish a consistent comparison across different examinations and different eyes, we created atrophy maps reporting FAF atrophy over an array of superpixels. The grid enabled us to represent landmark retinal locations, such as the fovea and the optic disc, on common positions for all eyes included (Fig. 1). First, to bring the optic disc and the fovea on the same axis we applied a vertical shear transformation to the nasal half of the image. Shear transformations allow the vertical dimension of the image to be shifted by a given angular value, leaving the horizontal dimension unmodified. After transformation, we constructed the superpixel grid centered on the fovea (location [0, 0]). Each superpixel was composed of a number of individual pixels in a *n by n* pixel configuration, and the dimension was chosen in each individual eye to achieve a fixed number of superpixels (20) separating the fovea and optic disc.^26^^,^^27^ The small variations of superpixel dimensions across different eyes allowed us to control for the varying fovea-optic disc distance across individuals and therefore account for anatomic variability. Each superpixel had the proportion of atrophic pixels within the superpixel as value, and the superpixel was considered atrophic when at least 50% of pixels were atrophic (i.e. value ≥ 0.5).

To assess the effect of age on the spatial progression of FAF atrophy, we used the atrophy maps described above to consistently relate examinations from different patients over a common spatial arrangement. We stratified all FAF atrophy maps by age at examination, starting from age 55 years, in 5 years strata (0–55, 55–60, 60–65, 65–70, and >70 years). Both 33 degrees and 55 degrees field FAF were included for this analysis, and locations where less than 4 exams were available were censored from analysis.

### OCT Images

We only considered OCT scans with acceptable quality (i.e. no eye movement or blink artifacts or media opacity), and if centration on the fovea could be ascertained. Disease severity in OCT scans was first assessed qualitatively, across the fovea B scan at baseline visit. The grading was based on recognized LORD changes in OCT scans and considered the following^9^: (1) intact RPE and EZ; (2) subretinal deposits; (3) disruption of RPE and EZ; and (4) severe outer retina atrophy. Complications such as epi-retinal membranes, choroidal neovascularization, and cystoid macular edema were also recorded.

Quantitative OCT metrics included in this study were the EZ width and retinal thickness at the fovea of the whole retina and the outer retina. These were measured by a senior clinician (author R.C.) considering the horizontal foveal B-scan displaying the foveal depression, the thickest outer nuclear layer, and the least residual inner retinal tissue. EZ width was manually measured as the sum of nasal and temporal widths, identified by the horizontal distance between the first EZ interruption in either side of the fovea at which EZ and RPE could not be differentiated. Measurements were performed with a semi-automated custom algorithm written in MATLAB (version 9.6.0; The MathWorks Inc., Natick, MA, USA). As suggested before,^28^ we considered breaks of the EZ only clear EZ loss, and an attenuation of the EZ signal alone was not considered such. Retinal thickness parameters were manually measured at the fovea, using the Spectralis’ inbuilt software. Central retinal thickness (CRT) considered the whole retina and was taken between inner limiting membrane and posterior border of the RPE-Bruch's membrane. We also extracted the thickness of the photoreceptor and RPE (PR+RPE) complex,^29^^,^^30^ taken between external limiting membrane and the posterior border of the RPE-Bruch's membrane. For consistency across scans and to avoid mismatch between axial and lateral resolutions, we considered thickness as the vertical distance between retinal layers of interest at the fovea.^31^

### Statistical Analysis

Given the relatively small sample size, quantitative clinical measures were extracted at all visits from both eyes of each participant. In addition, median and interquartile range (IQR) were adopted as summary statistics, and nonparametric statistics were used throughout the study. All statistical analyses were performed in *R*,^32^ and statistical significance was considered when *P* < 0.05.

Measures of BCVAs were transformed to LogMAR and values of counting fingers, hand movement, light perception, and no light perception, were converted to 2.6, 2.7, 2.8, and 2.9 LogMAR, respectively.^29^^,^^33^

Longitudinal changes of quantitative clinical measures were assessed by using only data from patients with two or more visits. We used linear mixed models to account for repeated measures from the same participant and to control for the inclusion of both eyes.^34^ For each quantitative measure, we built separate models; the clinical parameter of interest (e.g. CRT) was set as the outcome measure, age at visit was passed as fixed effect, and patient ID and eye laterality were passed as random effects (intercept). BCVA scores of counting fingers or worse represent end-stage disease with limited utility as an outcome metric in a trial setting,^35^ and were excluded from progression analysis. Where relevant, inflation points of longitudinal data series were estimated with piecewise linear regression using the package “segmented” in *R*.^36^ Interocular relationships of quantitative clinical measures were assessed using data from all visits, and we used repeated measure correlation to account for repeated measures from the same patient.^37^ Similarly, the relationship between different structural and functional parameters were tested with repeated measure correlation considering data at all visits.^37^ Family-wise Bonferroni correction was applied to control for multiple testing.

## Results

Sixteen patients with *C1QTNF5* mutations from 10 unrelated families were included from the Moorfields Eye Hospital database. Details of demographic characteristics of this cohort are reported in Supplementary Table S1. There were 10 female subjects (62.5%) in this sample, and the majority of patients were of White British ethnic background (*n* = 11, 68.8%). The remainder of the patients were of Greek Cypriot and Egyptian ethnicity (see the Table). Long zonules were found in most patients (*n* = 12, 80%). Difficulties with night vision were described as the presenting symptom by all patients, ranging from slower adaptation to impaired central night vision and significant difficulties with navigation. Symptoms were reported at the median age of 54 years (IQR = 43.8 to 56.5).

**Table.** Details of Genetic Variants of the *C1QTNF5* Gene Identified in This Study Population (Based on NM_015645.5)

#	Variant and Predicted Protein Change	Variant Type	Family ID	Gender	Ethnicity	BCVA at (Age)
01	Heterozygous c.489C>A, p.(Ser163Arg)	Missense	26573 – 1	Female	Egyptian	R: 0.0; L: 0.2 (61 y)
02	Heterozygous c.489C>G, p.(Ser163Arg)	Missense	4192 – 1	Male	White British	R: HM; L: HM (70 y)
			5062 – 1	Female	White British	R: 0.0; L: −0.05 (43 y)
			18176 – 1	Male	White British	R: −0.08; L: −0.08 (49 y)
			18176 – 2	Female	White British	R: 0.8; L: 1.0 (62 y)
			18176 – 3	Female	White British	R: 0.3; L: CF (64 y)
			18176 – 4	Male	White British	R: 1.5; L: 1.5 (72 y)
			19139 – 1	Male	White British	R: 0.3; L: 1.0 (64 y)
			19177 – 1	Female	White British	R: 0.8; L: 0.8 (71 y)
			20064 – 1	Female	White British	R: −0.06; L: 0.0 (59 y)
			26460 – 1	Male	White British	R: 0.02; L: −0.1 (58 y)
			26460 – 2	Female	White British	R: 0.22; L: 0.02 (61 y)
03	Heterozygous c.562C>A, p.(Pro188Thr)	Missense	28108 – 1	Female	Unknown	Unknown
04	c.556C>T, p.(Pro186Ser) c.569C>G, p.(Ser190Trp)*	Missense	19179 – 1	Female	Greek Cypriot	R: 0.5; L: −0.1 (54 y)
			19179 – 2	Male	Greek Cypriot	R: HM; L: HM (65 y)
			19179 – 3	Female	Greek Cypriot	R: 0.3; L: 0.3 (67 y)

Individual patients are described by their family identifier (GC number), gender (F: female, M: male), ethnicity, and best corrected visual acuity (BCVA, LogMAR) at their first visit. The corresponding age is reported in brackets. CF, counting fingers; HM, hand motion. *Variants are in cis.^18^

### Molecular Characteristics

Four genetic variants were identified in this cohort (see the Table), and all were previously reported. Most patients presented with the most common missense mutation c.489C>G p.(Ser163Arg), affecting 12 patients in 7 unrelated families. One patient presented with c.489C>A resulting in the same amino acid change p.(Ser163Arg), and 1 patient had c.562C>A, p.(Pro188Thr). There were 3 siblings with the heterozygous variants c.569C>G, p.(Ser190Trp), and c.556C>T, p.(Pro186Ser).

### Multimodal Imaging

**Figure 2.**
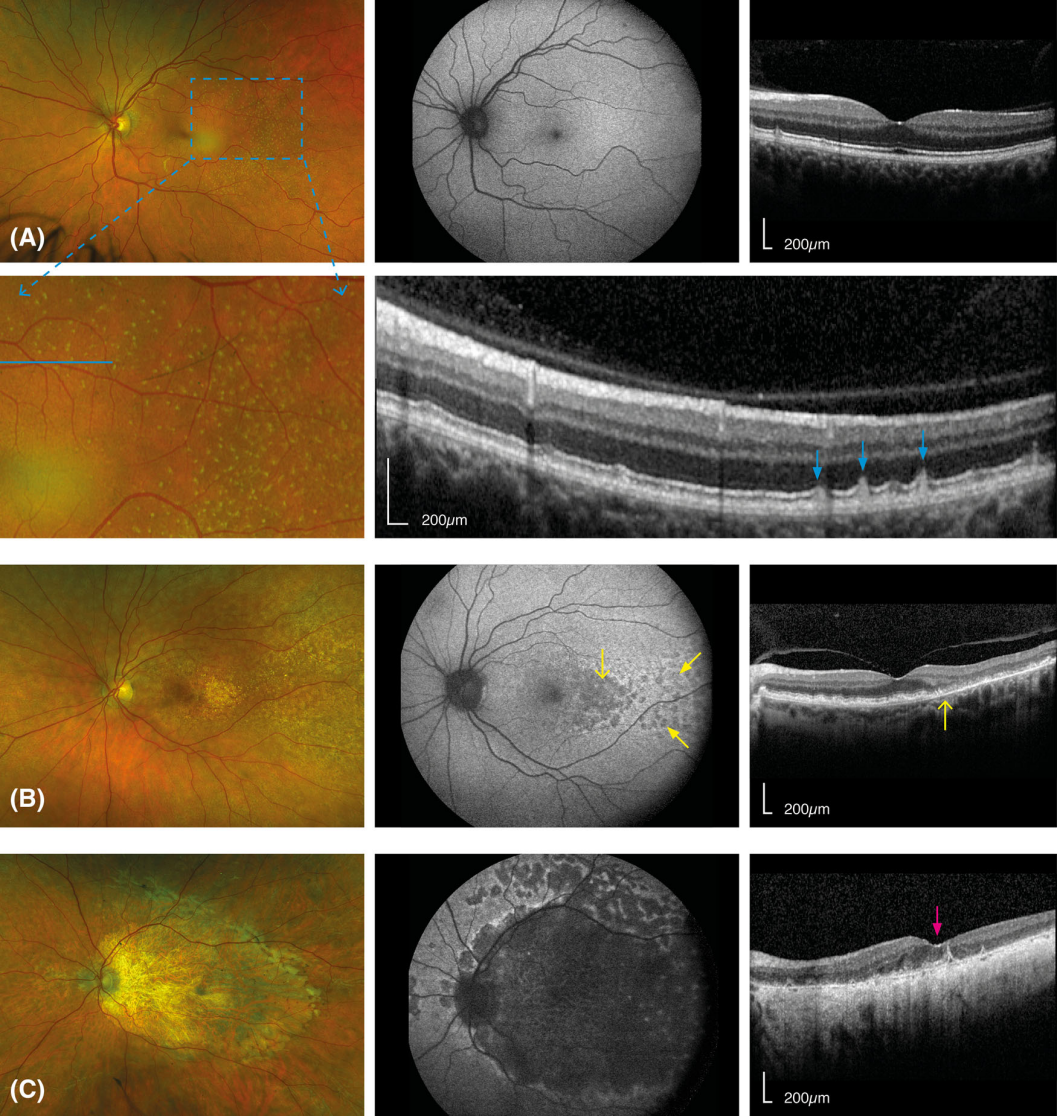
Multimodal imaging of representative patients with LORD from this cohort. (**A**) The 47 year old patient showing an intact macula and peri-macular yellow-white round lesions in the temporal retina (*blue square*). Fundus autofluorescence (FAF; *middle column*) and OCT foveal (*right-end side column*) scan also appear largely normal. An enlarged view of the yellow-white round lesions is reported in the *lower panel* of (**A**) visible as localized subretinal deposits in OCT scan resembling reticular pseudo-drusen (*blue arrows*). (**B**) The 63 year old patient showing an area of atrophy in the temporal retina. This is also visible in FAF, as a large hypofluorescence in the temporal macula (*yellow arrow*), surrounded by smaller focal changes (*yellow filled arrows*). The OCT fovea scan shows the transition between the relatively spared central macula including the fovea (*left hand-side of the yellow arrow*) and damaged temporal macula with severe atrophy of the outer retina (*right hand side of the yellow arrow*). (**C**) Patient with advanced LORD (age 72 years), exhibiting severe atrophy at the posterior pole in fundus photograph, consistent with a complete loss of autofluorescence. OCT foveal scan also shows severe atrophy of the outer retina and RPE involving the fovea (*pink arrow*). Hyper-transmission and choroid atrophy can also be observed across the whole b-scan.

Examples of multimodal imaging in representative patients with LORD are reported in Figure 2. Fundus photographs were available in 24 eyes from 12 patients, with a median age of 64.7 years (IQR = 57.9 to 69.4) at baseline. Only 4 eyes (16.7%) presented at LORD stage 2, whereas the majority presented the most severe stage 3 with atrophy at the posterior pole (*n* = 20, 83.3%; see Supplementary Table S1). The right and left eyes from each patient showed consistent severity stage. Overall, eyes at stage 2 were younger (47.8 years, IQR = 46.4 to 49.3 vs. 66.4 years, IQR = 61.2 to 70.0, *P* = 0.002) and had better BCVA (−0.04 LogMAR, IQR = −0.08 to −0.05 vs. 0.24 LogMAR, IQR = 0.2 to 2.7, *P* = 0.036) compared to eyes at stage 3.

### Fundus Autofluorescence-Related Atrophy

There were 31 eyes from 16 patients with at least one gradable FAF image at a median baseline age of 65.2 years (IQR = 59.1 to 67.1 years), and most eyes demonstrated atrophic changes at their first visit (*n* = 24, 77.4%; see Supplementary Table S1). Eyes without FAF atrophy had better BCVA (−0.03 LogMAR, IQR = −0.09 to 0 vs. 1.3 LogMAR, IQR = 0.2 to 2.7, *P* = 0.0007) and younger age (54.7 years, IQR = 46.6 to 58.3 vs. 65.6 years, IQR = 63.8 to 68.5, *P* = 0.0003) compared to those with atrophic changes.

**Figure 3.**
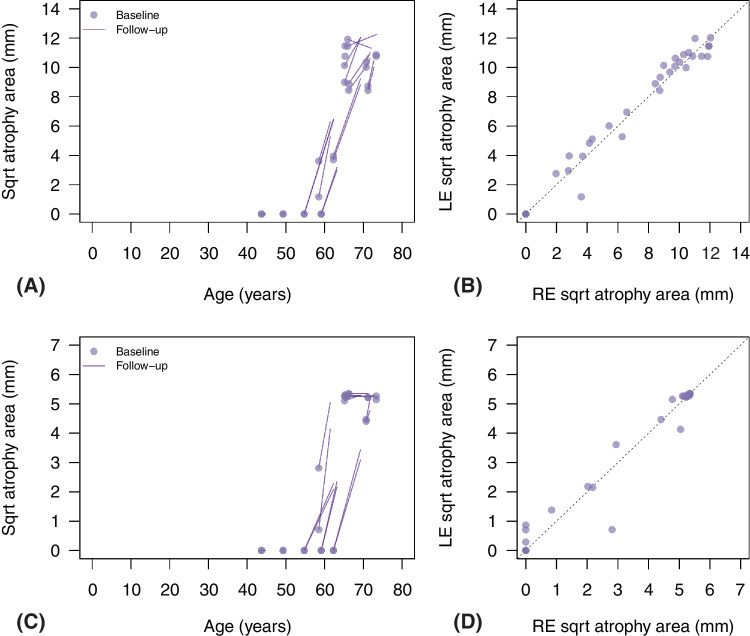
Changes in square root (sqrt) area of atrophy (AA) in fundus autofluorescence (FAF) images by age (**A, C**) and interocular relationship (**B, D**). In (**A**), points report sqrt AA within 14 mm diameter circle at baseline for all eyes. For eyes with follow-up examinations, data at all visits were fitted with linear regression and segments report the corresponding fit for each eye. (**B**) Interocular relationship for sqrt AA within the 14 mm diameter circle at all visits. A similar representation is reported for sqrt AA within the 6 mm diameter circle, with changes over time in panel (**C**) and inter-ocular relationship in panel (**D**).

Analysis of FAF AA was limited to 55 degrees field images, which were available in 26 eyes from 13 patients at least at one visit (median baseline age = 65.2 years, IQR = 58.5–66.2 years). There were 23 eyes from 12 patients with 2 visits or more (median = 3 and maximum = 6), with median follow-up of 4.0 years (IQR = 1.5 to 6.1 years). Median AA at baseline was 19.7 mm^2^ (IQR = 0 to 27.6) and 71.1 mm^2^ (IQR = 0 to 106.2) within 6 mm and 14 mm diameter, respectively. Age related changes are shown in Figure 3, and we found a significant effect of age on square root AA when considering both circle diameters (χ^2^ = 48.5 and 79.6, both *P* < 0.0001). Progression rates were + 0.24 mm/year (95% confidence interval [CI] = 0.18–0.29, *R*^2^ = 0.657, *P* < 0.0001), and + 0.53 mm/year (95% CI = 0.45–0.62, *R*^2^ = 0.774, *P* < 0.0001) for the 6 mm and 14 mm diameter circle, respectively. As evidenced in Figure 3, there were no detectable atrophic changes until age 50 to 60 years, at which point a steady AA progression was noted. The estimated inflection point (piecewise linear regression) was 56.3 years (95% CI = 52.5–60.1) and 53.9 years (95% CI = 45.9–61.8) for the 6 mm and 14 mm diameters circle, respectively. Interocular correlation for square root AA is reported in Figure 3, and we found a strong relationship for the 6 mm (*r* = 0.97, 95% CI = 0.92–0.99, *P* < 0.0001), and the 14 mm diameter circle (*r* = 0.90, 95% CI = 0.76–0.96, *P* < 0.0001).

**Figure 4.**
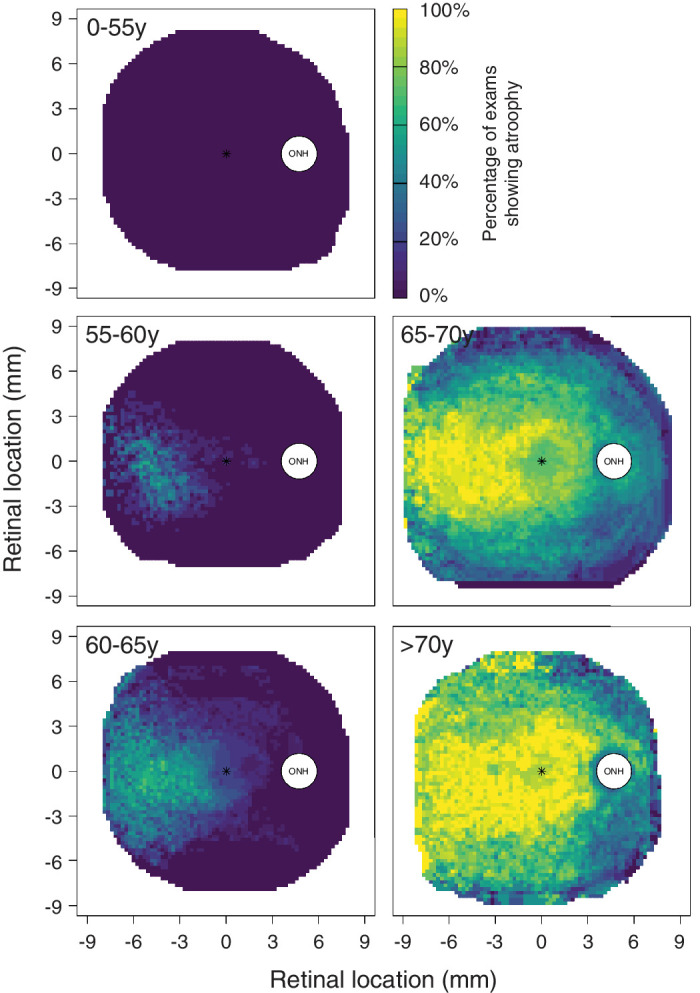
Spatial pattern of progression of fundus autofluorescence (FAF) atrophy by age. Heat-maps for different age strata are based on all examinations available within the given age group. The color at each location is attributed based on percentage of examinations showing atrophy at that location among all examinations from the specific age-group. Only locations with four or more examinations are plotted. Fovea location is reported by (*) in each map. Group 0 to 55 years (*n* = 11 examinations), group 55 to 60 years (*n* = 12 examinations), group 60 to 65 years (*n* = 20 examinations), group 65 to 70 years (*n* = 31 examinations), and group > 70 years (*n* = 21 examinations).

A few patients in this cohort had available follow-up data with baseline examinations showing no or minimal FAF atrophy. Findings in these eyes are reported in Supplementary Figure S1, and suggested that atrophy appeared initially in the temporal retina. To better assess such a progression pattern, we grouped all FAF atrophy maps by age strata starting from age 55 years, in 5-year steps. There were 95 usable FAF images for this analysis from 30 eyes of 15 patients (23 examinations with 30 degrees field, and 72 examinations with 55 degrees field). Findings are reported in Figure 4, and the heat-maps describe the percentage of examinations showing atrophy at each location across different age groups. This analysis suggests that FAF atrophy often becomes visible at first in the temporal retina between age 55 and 60 years, before progressing to the whole posterior pole with extensive atrophy from age 65 years and older.

### Spectral Domain-Optical Coherence Tomography

There were 28 eyes from 15 patients with at least one usable OCT examination (median baseline age = 64.1 years, IQR = 58.7–67.0 years), and the majority presented with some degree of outer retina atrophy at baseline (*n* = 23, 82.1%). Neither cystoid macular edema nor choroidal neovascularization was noted at baseline, whereas 3 eyes had epiretinal membranes (10.7%). Eyes with atrophy frequently showed highly disorganized outer retina and subretinal hyper-reflective changes (*n* = 8, 28.6%). Follow-up examinations were available in 21 eyes of 11 patients (median = 5 visits and maximum = 7), with a median follow-up of 6.4 years (IQR = 2.7–8.6 years).

Median EZ width at baseline was 1006 µm (IQR = 0–4288), and this parameter was significantly affected by age (χ^2^ = 92.9, *P* < 0.0001, Supplementary Fig. S2), with a progression rate of −257 µm/year (95% CI = −295.0 to −218.9, *R*^2^ = 0.792, *P* < 0.0001). Retinal thickness measures showed larger intersubject variability (see Supplementary Fig. S2), with median values at baseline of 226.5 µm (IQR = 190.3–249.5) and 93 µm (IQR = 19–107) for CRT and PR+RPE, respectively. Both thickness parameters also showed significant loss with age (χ^2^ = 43.7 and 47.8, both *P* < 0.0001) with progression rates of −4.7 µm/year (95% CI = −6.0 to −3.5, *R*^2^ = 0.370, *P* < 0.0001) and −3.8 µm/year (95% CI = −4.8 to −2.9, *R*^2^ = 0.477, *P* < 0.0001). Consistent with FAF AA, OCT metrics showed a dual progression pattern, without substantial changes until adulthood, followed by a steadier loss (see Supplementary Fig. S2). The estimated inflection point for EZ width was 50.8 years (95% CI = 43.4 to 58.2). No obvious thinning could be observed for CRT and PR+RPE until around 10 years later than observed with EZ width, with inflection points of 62.3 years (95% CI = 58 to 66.5) and 60 years (95% CI = 55.2 to 64.8) for CRT and PR+RPE, respectively. Interocular relation for OCT parameters is reported in Supplementary Figure S2, and we found strong correlation for both EZ width (*r* = 0.90, 95% CI = 0.81 to 0.95, *P* < 0.0001), and CRT (*r* = 0.85, 95% CI = 0.71 to 0.92, *P* < 0.0001). Slightly poorer interocular correlation was found for PR+RPE (*r* = 0.65, 95% CI = 0.41 to 0.81, *P* < 0.0001).

**Figure 5.**
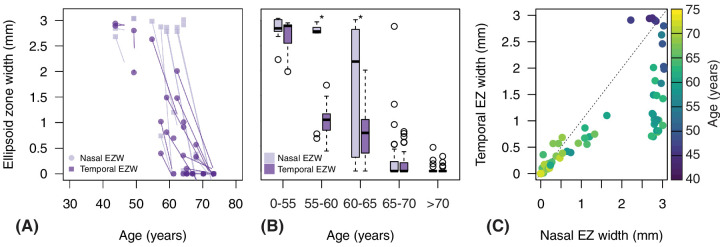
Differences between temporal and nasal ellipsoid zone (EZ) widths. In (**A**) changes of EZ width by age are reported for nasal and temporal EZ width. The plotting is consistent with Supplementary Figure S2. (**B**) Reports boxplots of the difference between nasal and temporal EZ width for different age groups, and the same strata adopted in Figure 4 are considered. Statistical significance was considered when *P* < 0.05 and reported by (*). (**C**) Shows the scatterplot between nasal and temporal EZ width. Data are color coded by age at the time of examination.

In accordance with FAF atrophy, EZ width exhibited different disease appearance between its temporal and nasal components. As reported in Figure 5A, temporal EZ width seemed to start degenerating earlier than nasal EZ width. On average, temporal EZ width was 350 µm shorter than the nasal component (95% CI = 231 to 470, *P* < 0.0001), while controlling for age, repeated patient, and eye. Yet, as reported in Figure 5B, nasal and temporal EZ widths were similar until age 55 years and beyond age 65 years, and most differences could be noted between age 55 and 65 years (age group 55–60 = 1020 µm vs. 2780 µm, *P* = 0.019; age group 60–65 years = 760 µm vs. 2180 µm, *P* = 0.012; see Fig. 5B). Figure 5C reports the relationship between temporal and nasal EZ widths of the same eye, and it shows little change of the nasal EZ width until substantial loss of the temporal component has occurred.

### Best Corrected Visual Acuity 

Measures of BCVA were available in 30 eyes of 15 patients (median baseline age = 62.3 years, IQR = 58.8–65.4 years), and 28 eyes from 14 patients had 2 or more visits (median = 5.0) for a median follow-up time of 5.1 years (IQR = 2.6–7.6 years). Two eyes from two patients (age at baseline 65 and 55 years) were excluded from further analysis due to concurrent amblyopia and optic neuritis resulting in impaired BCVA before fovea involvement due to LORD. At baseline, median BCVA was 0.3 LogMAR (IQR = 0 to 1.0), dropping to a median of 1.4 LogMAR (IQR = 0.29 to 2.7) at the final visit. Longitudinal changes of BCVA are reported in Supplementary Figure S3 and we found a significant effect of age (χ^2^ = 39.9, *P* < 0.0001), with an estimated rate of loss of + 0.05 LogMAR/year (95% CI = 0.04 to 0.06, *R*^2^ = 0.448, *P* < 0.0001). BCVA remained largely preserved until late adulthood, and the estimated inflation point was 70.3 years (95% CI = 68.6 to 72 years). Consistently with other metrics, BCVA was highly correlated between the two eyes (*r* = 0.77, 95% CI = 0.63 to 0.86, *P* < 0.0001).

### Additional Analyses

**Figure 6.**
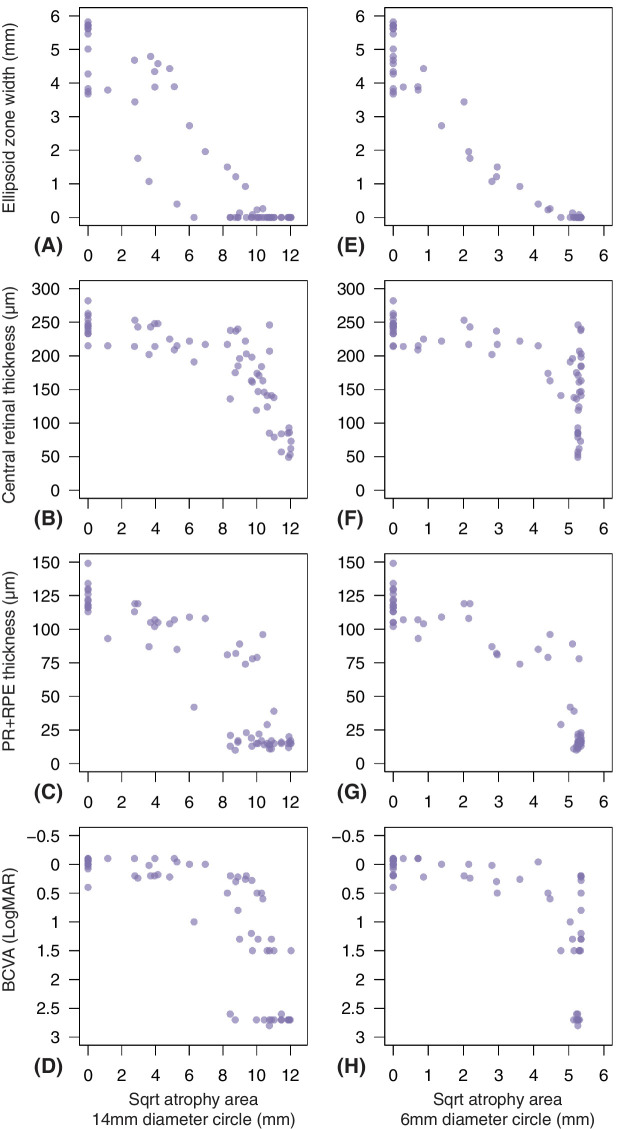
Scatterplots of the relationship between square root (sqrt) atrophy area (AA) within the 14 mm and 6 mm diameter circles and other clinical parameters. Relationship between sqrt AA and ellipsoid zone width (**A, E**), central retinal thickness (**B, F**), photoreceptor and retinal pigment epithelium complex (PR+RPE), (**C, G**) and best corrected visual acuity (BCVA) (**D, H**) were assessed by considering all visits.

Correlation between FAF AA and other clinical parameters is reported in Figure 6, and Supplementary Table S2. We found a strong negative correlation between square root AA and EZ width (*r* = −0.92 to −0.87), and poorer correlation was observed with retinal thickness measures (CRT: *r =* −0.18 to −0.39; and PR+RPE: *r* = −0.47 to −0.51). Similarly, BCVA and FAF AA were only moderately related (*r* = 0.30 to 0.34).

## Discussion

LORD is an inherited macular dystrophy that remains to be fully characterized. Our study reported a longitudinal analysis of retinal structural modifications in a cohort of patients with molecularly confirmed *C1QTNF5* heterozygous variants. There are still no therapies proven to arrest or delay LORD progression but there is increasing interest, and potential gene therapies or therapeutic avenues, such as Metformin^8^ and vitamin A.^19^ Our findings could provide improved guidance for forthcoming clinical trials and enhance the overall prediction of outcomes for patients with LORD.

Patients in our cohort presented symptomatic changes in night vision between age 45 and 55 years. This is consistent with previous studies conducted in samples with a comparable genotype, showing that nyctalopia was first reported in the mid 40s by patients with the p.(Ser163Arg) mutation.^38^^,^^39^ Similarly, in a cohort of 26 patients harboring the p.(Pro188Thr) mutation, age of symptoms onset was around 50 years, ranging between 35 and 56.^10^ After the onset of symptoms, atrophic changes of the outer retina and the RPE were absent until age 50 to 55 years in our patients, and then quickly progressed. By age 70 to 75 years most patients showed advanced atrophy and severe central visual impairment.

### Considerations for Future Trials

Our longitudinal analysis showed square root AA in FAF images and EZ width to be promising outcome metrics for LORD monitoring, with detectable changes at or short after age of symptoms onset (age 50.8 to 56.3 years), rapid progression rates (4.3% to 4.5%, rates normalized for their dynamic ranges), and strong interocular correlation (*r* = 0.90 to 0.97). Manual mapping of area of geographic atrophy in FAF images and the derived mean rate of change are recognized outcome metrics in clinical trials for AMD.^24^^,^^40^^,^^41^ Considering LORD, due to the configuration of scalloped atrophy in FAF images, AA appears to be a robust and easily measurable metric.^14^^,^^15^ Measures of EZ width over the foveal B-scan also showed significant age-related loss and strong interocular correlation in our study. This is not surprising as EZ parameters have been established as robust outcome metrics for many inherited retinal diseases, as being a highly repeatable objective metrics and with excellent correlation to visual function.^42^^,^^43^

Previous small cohort studies have provided qualitative description of LORD clinical course,^9^^,^^10^^,^^16^^,^^17^^,^^38^ but only a few studies quantified age-related changes in this condition.^14^^,^^15^^,^^39^ To the best of our knowledge, there is no previous work assessing rate of change of EZ parameters in LORD, whereas a few studies have provided progression rates for AA in FAF images.^14^^,^^15^ A longitudinal study by Vanderford et al. included four LORD patients all harboring the p.(Ser163Arg) mutation.^14^ Among the 5 eyes which developed atrophy (30 degrees field FAF images) the square root AA progression rate ranged between 0.53 and 1.97 mm/year, being 0.55 mm/year in the one with longer follow-up.^14^ Another longitudinal study followed 16 patients with LORD with the p.(Ser163Arg) mutation for 3 years and yearly visits.^15^ Patients were only included in that study if foveal atrophy was not present in FAF images at first visit, and consistently with our study, authors manually mapped FAF atrophy from 55 degrees field images. At their first visits, patients had a mean (± standard deviation) total AA of 2.6 ± 4.7 mm^2^, which increased to 9.2 ± 8.4 mm^2^ at follow-up, with the progression rate of square root AA of 0.57 ± 0.34 mm/year. Patients in our cohort showed larger AA at baseline (median = 71.1 mm^2^, IQR = 0–106.2) compared to Borooah et al.^15^ This is not surprising given the slightly younger mean age (62.1 vs. 65.2 years) and their inclusion criteria that may have led to exclusion of patients with more advanced atrophy. Overall, the progression rates from available studies are highly consistent with our estimate from the 14 mm diameter circle (0.53 mm/year, 95% CI = 0.45 to 0.62), suggesting a quick progression rate of RPE atrophy.

Our results suggest that retinal thickness and BCVA may be poorer outcome metrics compared to EZ width and AA. These parameters appeared to be affected later (age 60–70 vs. 50.8–56.3) and showed less prominent age-related modifications (1.6–3.3% vs. 4.3–4.5%). Correlation with square root AA also showed retinal thicknesses and BCVA to remain largely stable for considerable AA extension with reduction only at high levels of AA (see Fig. 6). Furthermore, early and moderate LORD stages are characterized by subretinal deposition and RPE-Bruch's membrane separation.^13^ Retinal atrophy may, therefore, be masked by initial outer retina thickening. The work from Borooah et al.^15^ mentioned above also assessed changes in chorioretinal thickness and did not find significant differences over 3 years. Our longitudinal analysis is based on a similar sample size, but slightly longer follow-up and more advanced disease. Although we found significant retinal thinning with age, changes manifested later on in the disease course and were slower compared to EZ width and AA parameters. Together with previous studies,^13^^,^^15^ our results may question the suitability of outer retinal thickness metrics in LORD as they may exhibit multiple trends as the disease progresses. Similarly, BCVA may be a poorer outcome, as it is determined by intact foveal structure, which appeared preserved until late in many patients of our study. Previous studies have also showed BCVA to be well preserved at moderate LORD stages,^10^^,^^38^ and maintained to near normal levels until age 60 years. Clinically meaningful and rapid decline is often observed only after age 65 years.^10^

All clinical parameters assessed in this study showed high interocular correlation, suggesting symmetric disease presentation and progression. This is in agreement with previous studies, which also showed strong correlation for areas of FAF lesions and chorioretinal thicknesses.^14^^,^^15^ Accordingly, study designs administering treatment to only one eye and using the fellow eye as control may be considered. Concerning a potential treatment window, patients with LORD exhibited symptoms from age 45 years, and EZ width and FAF AA were rapidly progressing from age 50 and 55 years, respectively. Trials recruiting patients in their mid 50s should ensure treatment is administered long before central macular structure and function are fully lost and clinical metrics may show rapid change enabling timely trial completion.

We found both FAF atrophic changes and EZ loss to originate temporally (see Figs. 4, 5), and this is consistent with several previous studies showing temporal presentation and nasal progression of atrophy in patients with the p.(Ser163Arg) mutations.^9^^,^^14^^,^^15^^,^^17^^,^^38^^,^^39^ More variability in the pattern of disease progression may be observed in case of different mutations, such as the p.(Pro188Thr).^10^ A study on 16 patients with LORD and p.(Ser163Arg) mutations showed 61.3% and 32.2% of patients presenting atrophy in the outer ETDRS temporal and nasal sectors, respectively.^15^ This progression pattern may be a characteristic feature of LORD and remains unexplained. A study on choroidal neovascularization in four patients with LORD found predilection for membranes to present in the superior and inferior quadrants, leading authors to raise a potential role for choroidal watershed zones.^44^ A later study, however, did not find localization of reticular pseudo-drusen in correspondence of choroidal lobules.^16^ Early work in LORD assessed subretinal deposits from one donor eye,^3^ and found some sectorial variation, with thicker lipids in the mid-periphery compared to the macula, and thicker depositions associated to more severe photoreceptor loss. Yet, size variation of retinal deposits does not fully explain predilection for initial temporal presentation of RPE atrophy. Other authors have considered the potential implications of C1QTNF5 dysfunction in rods metabolism.^15^ The protein is part of C1q, which represents the first subcomponent of the C1 complex, with important roles in glucose regulation and fatty acid oxidation. The temporal mid periphery of the retina has high density of rods, whose metabolism strongly relies on glucose transport from choroid.^45^ It has been hypothesized that subretinal deposition and impaired transport from choroid could be particularly detrimental to rod photoreceptors in this region of high metabolic demand. In contrast, other retinal cell populations in different regions of the retina, such as Müller glia in the fovea, could compensate for this metabolic dysfunction enabling a delayed or slower degeneration.^15^

In our cohort, changes in EZ width seemed to occur earlier than FAF atrophy (age 51 vs. 54 years). This is especially true considering the temporal onset of atrophy and the larger field captured by FAF (55 degrees) compared to only the central 20 degrees of our macular OCT scans. An OCT examination based on wider-field may capture EZ loss earlier than the current parameters. Our correlation analysis further supports earlier EZ changes (see Fig. 6), with qualitative observation of scatter plots between square root AA and EZ width showing little AA change at higher EZ width ranges. Similar results were proposed in a prospective case series of 3 patients with LORD examined with multimodal imaging and mesopic fundus-tracked perimetry over 4 years.^39^ Although areas of EZ loss in OCT scans were associated with rod functional impairment, there was poorer correspondence with FAF lesions, and areas of speckled autofluorescence provided better relationship with EZ loss than atrophy. As emphasized by previous LORD natural history studies, atrophy is a late finding in FAF imaging, and there are earlier modifications detectable in this imaging modality.^9^^,^^13^^–^^17^ The longitudinal study by Vanderford et al. described above^14^ considered lesions in both OCT and FAF and estimated that FAF may show a transition from reticular pseudo-drusen to speckled autofluorescence 8 to 10 years before atrophy appears. Overall, our study consolidates results from previous small case series suggesting that EZ width may be an earlier marker of LORD progression. RPE atrophy as measured in FAF may be a less sensitive metric, because changes appear later on in the disease course.^14^^,^^39^ Previous reports agree that speckled FAF anticipates atrophy and may be more consistent with EZ loss,^14^^,^^39^ but arguably this lesion may be more challenging to be quantified in a clinical trial setting where objective and highly repeatable metrics are desirable. Segmenting areas of speckled changes in FAF may be a subtler and less repeatable task compared to detection of atrophy, and evidence from AMD studies showed much lower agreement when detecting increased pigmentation in fundus photographs compared to geographic atrophy.^46^^,^^47^

### Limitations

LORD is a rare retinal dystrophy and although our study is among the largest longitudinal case series in this literature, the sample size is small, with consequent limitations of statistical power and sample heterogeneity. Accordingly, most of our patients (75%) presented with the mutation p.(Ser163Arg), and we could not perform genotype-phenotype correlation analysis. In addition, owing to the small sample size, within the analysis of FAF progression pattern (see Fig. 4) we allowed for more than one image from the same patient to be included within a given age group, in case of follow-up examinations available. This may have led to patients with longer and more frequent follow-up to be more largely represented in this analysis. Data for this study were retrospectively collected from routine hospital visits, and this may have resulted in variable test type and frequency of examinations between patients. Accordingly, some participants were imaged with as little as 19 B-scans per OCT examination, with the chance that focal EZ changes may have remained undetected. For similar reasons, our FAF analysis was limited to 55 degrees field images due to imaging availability rather than ideal field of visualization. Indeed, patients with LORD may present atrophic changes beyond 55 degrees in their later stages, and quantifiable wide field imaging may provide relevant information of disease progression. Last, FAF atrophy and OCT metrics were extracted by a single senior clinician. However, manual measures of AA are largely adopted and have shown excellent repeatability in AMD and LORD.^15^^,^^48^ Similarly, the measures of EZ width and retinal thickness considered herein are well established in the area of inherited retinal diseases, with accepted inter- and intra-observer reliability.^31^^,^^43^^,^^49^ Nonetheless, methods allowing automated data extraction may improve the data collected.

## Conclusions

Patients with LORD remain asymptomatic until age 45 to 50 years, and symptom onset is shortly followed by EZ loss and RPE atrophy. Both EZ width and area of atrophy in FAF images show rapid progression rates and high interocular correlation, representing promising outcome metrics in LORD. Clinical parameters reveal a dramatic progression rate, with most patients showing complete outer retinal atrophy and severe visual impairment by age 75 years. For most of our patients, EZ loss and RPE atrophy often originated temporally. Accordingly, clinical measures also capturing temporal retina may be preferable, as they are capable of detecting earlier changes and may enable monitoring of patients for a longer period of time.

## Supplementary Material

Supplement 1
