# Supplementary material for: Longitudinal Changes of Retinal Structure in Molecularly Confirmed C1QTNF5 Patients With Late-Onset Retinal Degeneration
Source: Transl Vis Sci Technol. 2023 Dec 12;12(12):14. doi: 10.1167/tvst.12.12.14 (PMC10720756; doi:10.1167/tvst.12.12.14)
Supplement: Supplement 1 [file tvst-12-12-14_s001.pdf]

SUPPLEMENTARY TABLES

Supplementary Table 1. Clinical characteristics and demographics of patients with *C1QTNF5*-LORD in this study. Continuous variables are reported as median and interquartile range (IQR); categorical variables as count (n) and frequency (%).

	n or median	% or IQR
**Gender** (female / male)	10 / 6	62.5% / 37.5%
**Ethnicity**		
White British, n (%)	11	68.8%
Greek Cypriot, n (%)	3	18.8%
Egyptian, any, n (%)	1	6.2%
Unknown	1	6.2%
**Age at first symptoms** (n=15) Median (IQR), years	54	43.8-56.5
**Presenting symptoms** (n=15)		
Night Blindness, n (%)	15	100.0%
**Age at first visit** Median (IQR), years	62.3	58.8-65.4
**Follow-up time** Median (IQR), years	5.1	2.6-7.6
**BCVA baseline, RE** (n=30 eyes) Median (IQR), LogMAR	0.3	0.0-1.0
**BCVA last visit, RE** (n=28) Median (IQR), LogMAR	1.4	0.29-2.7
**Fundus photo baseline (Borooah al stages)** (n=24 eyes)		
Stage 1, n (%)	0	0%
Stage 2, n (%)	4	16.7%
Stage 3, n (%)	20	83.3%
**FAF baseline**		
Qualitative (n=31 eyes)		
Normal FAF, n (%)	0	0%
Speckled FAF or reticular pseudo-drusen, n (%)	7	22.6 %
Atrophic FAF changes, n(%)	24	77.4%
*Quantitative at baseline (*n=26 eyes*)*		
FAF atrophy area, 6mm diameter circle, mm^2	19.7	0-27.6
FAF atrophy area, 14mm diameter circle, mm^2	71.1	0-106.2
**OCT metrics baseline**		
Qualitative (n=28 eyes)		
Intact EZ & RPE, n (%)	0	0%
Sub-retinal deposits and pseudo-drusen, n (%)	5	17.9%
Disruption of RPE and EZ in macula, n (%)	14	50%
Severe atrophy, n (%)	9	32.1%
Quantitative at baseline (n=28 eyes)		
EZ width, μm	1006	0-4288
CRT, μm	226.5	190.3-249.5
PR+RPE thickness, μm	93	19-107
**Long anteriorly inserted zonule,** (n=15)		
Present, n (%)	12	80%
Absent, n (%)	1	6.7%
Pseudophakic at first visit, n (%)	2	13.3%

BCVA: best corrected visual acuity; RE: right eye; FAF: fundus autofluorescence; EZ: ellipsoid zone; CRT: central retinal thickness; PR+RPE: photoreceptor and retinal pigment epithelium complex.

**Supplementary Table 2.** Correlation between square root FAF area of atrophy and other clinical parameters assessed in this study. For each parameter we report the correlation coefficient, the 95% confidence interval (within brackets), and the corresponding p value measured with repeated measure correlation, see methods. After Bonferroni correction (8 comparisons, p=0.006), significant relationships are flagged with *. EZ = Ellipsoid zone; CRT = Central retinal thickness; PR+RPE = Photoreceptor and retinal pigment epithelium complex; VA = Best corrected visual acuity.				
	**EZ width**	**CRT**	**PR+RPE**	**BCVA**
**Square root FAF atrophy area, 6 mm diameter circle**	-0.92<br>(-0.96 to -0.87)<br>p<0.0001*	-0.18<br>(-0.44 to 0.1)<br>p=0.19	-0.47<br>(-0.66 to -0.22)<br>p=0.0003*	0.30<br>(0.02 to 0.54)<br>p=0.034
**Square root FAF atrophy area, 14 mm diameter circle**	-0.87<br>(-0.93 to -0.79)<br>p<0.0001*	-0.39<br>(-0.60 to -0.13)<br>p=0.004*	-0.51<br>(-0.69 to -0.28)<br>p<0.0001*	0.34<br>(0.06 to 0.57)<br>p=0.015

Supplementary Table 3. OCT scans and fundus autofluorescence (FAF) images collected in participants of this study. Macular cube scans were acquired in all participants and the size of the cube in degrees is reported. N of B-Scan refers to the number of b lines comprising each cube scan. Vis (n) refers to the total number of visits with available OCT scans for the specific patient and eye. By using the follow-up setting within the Spectralis OCT, baseline OCT scans parameters were preserved at follow-up examinations. FAF RE and FAF LE report the number of FAF images available with the 55° and 30° field that could be used for quantitative analyses of atrophy. When both 30° and 55° images were available and usable at a given visit, the 55° only was selected. One patient (ID: 26460 – 2) had wide field FAF images that could be used for qualitative grading but were discarded for quantitative analysis for consistency.

**Family ID**	**OCT RE**			**OCT LE**			**FAF RE**		**FAF LE**	
	Pattern	N of B-Scan	Vis (n)	Pattern	N of B-Scan	Vis (n)	55° (n)	30° (n)	55° (n)	35° (n)
26573 – 1	cube_20_15	19	7	cube_20_15	19	7	3	1	4	0
4192 – 1	cube_20_20	25	3	cube_20_20	25	3	2	1	2	1
5062 – 1	cube_20_20	49	3	cube_20_20	49	3	2	1	2	1
18176 – 1	cube_20_20	25	2	cube_20_20	25	2	2	0	1	0
18176 – 2	-	-	-	cube_20_20	25	1	1	0	1	0
18176 – 3	cube_20_20	25	5	cube_20_20	25	5	3	0	3	0
18185 – 1	-	-	-	-	-	-	0	1	0	1
19139 – 1	cube_20_20	25	7	cube_20_20	25	7	5	0	4	0
19177 – 1	cube_20_15	19	1	cube_20_15	19	1	1	0	1	0
20064 – 1	cube_20_15	19	7	cube_20_15	19	7	3	4	3	4
26460 – 1	cube_20_20	49	3	cube_20_20	49	3	2	1	2	1
26460 – 2	cube_30_20	49	1	cube_30_20	49	1	-	-	-	-
28108 – 1	cube_20_20	25	1	cube_20_20	25	1	0	1	0	1
19179 – 1	-	-	-	cube_20_20	49	6	6	0	6	0
19179 – 2	cube_20_20	25	6	cube_20_20	25	6	4	0	5	0
19179 – 3	cube_20_15	19	5	cube_20_15	19	4	2	2	2	2

SUPPLEMENTARY FIGURES

Supplementary Figure 1

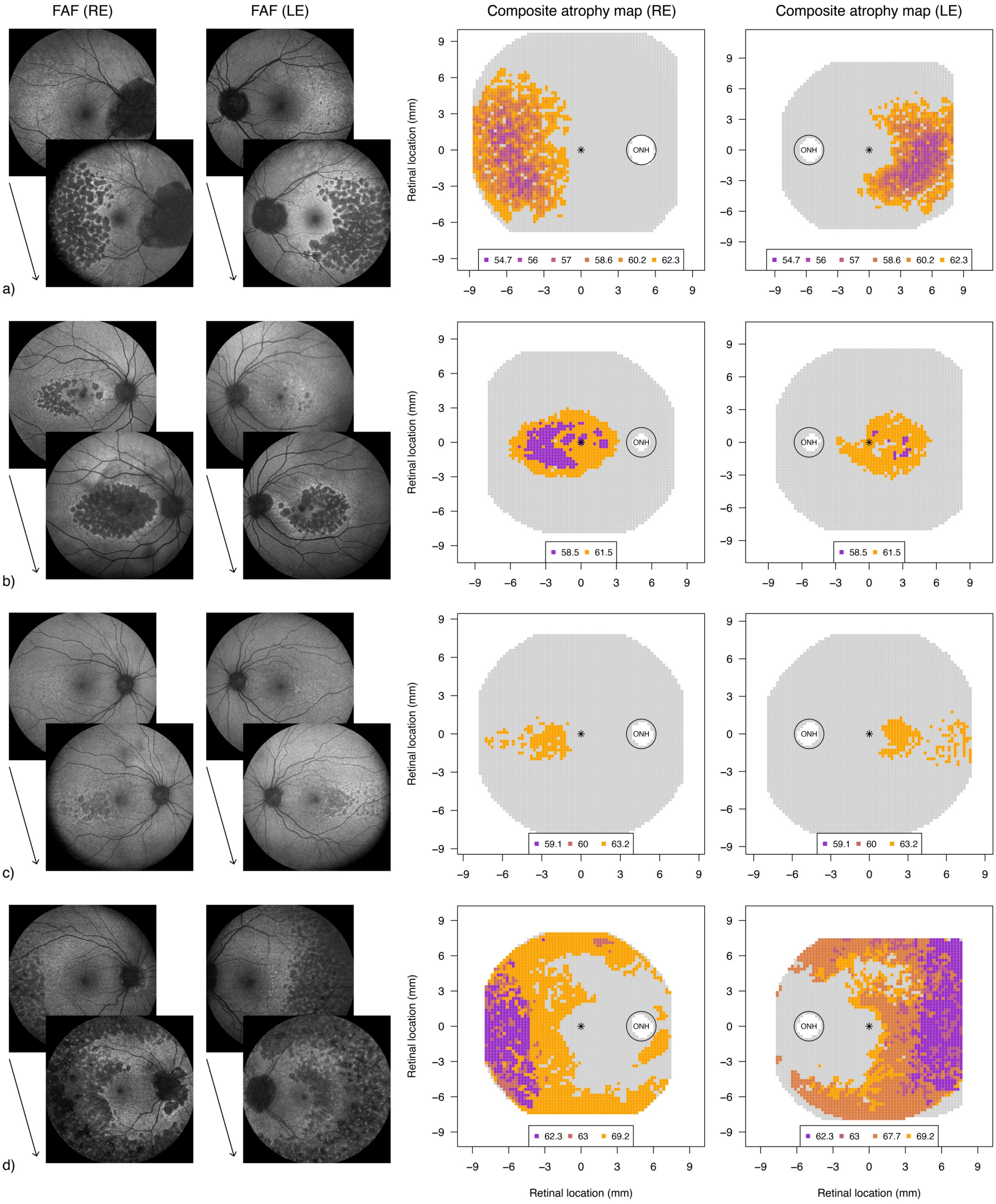

Supplementary Figure 1. Fundus Autofluorescence (FAF) images at baseline and last visit for 4 patients with LORD showing no or limited areas with atrophy at their baseline visits. Findings from manual mapping of atrophy at all visits are reported in a composite atrophy map where new areas of atrophy are reported for each follow-up visit and colour coded accordingly. Age at the examination is reported in the legend. Patients are represented with increasing age at baseline FAF and are the following, a) GCN 19179, heterozygous mutation c.569C>G; c.556C>T (p.Ser190Trp ; p.Pro186Ser); b) GCN 26460, mutation c.489C>G, (p.Ser163Arg); c) GCN 20064, mutation c.489C>G, (p.Ser163Arg); d) GCN 26573, mutation c.489C>A, (p.Ser163Arg).

Supplementary Figure 2

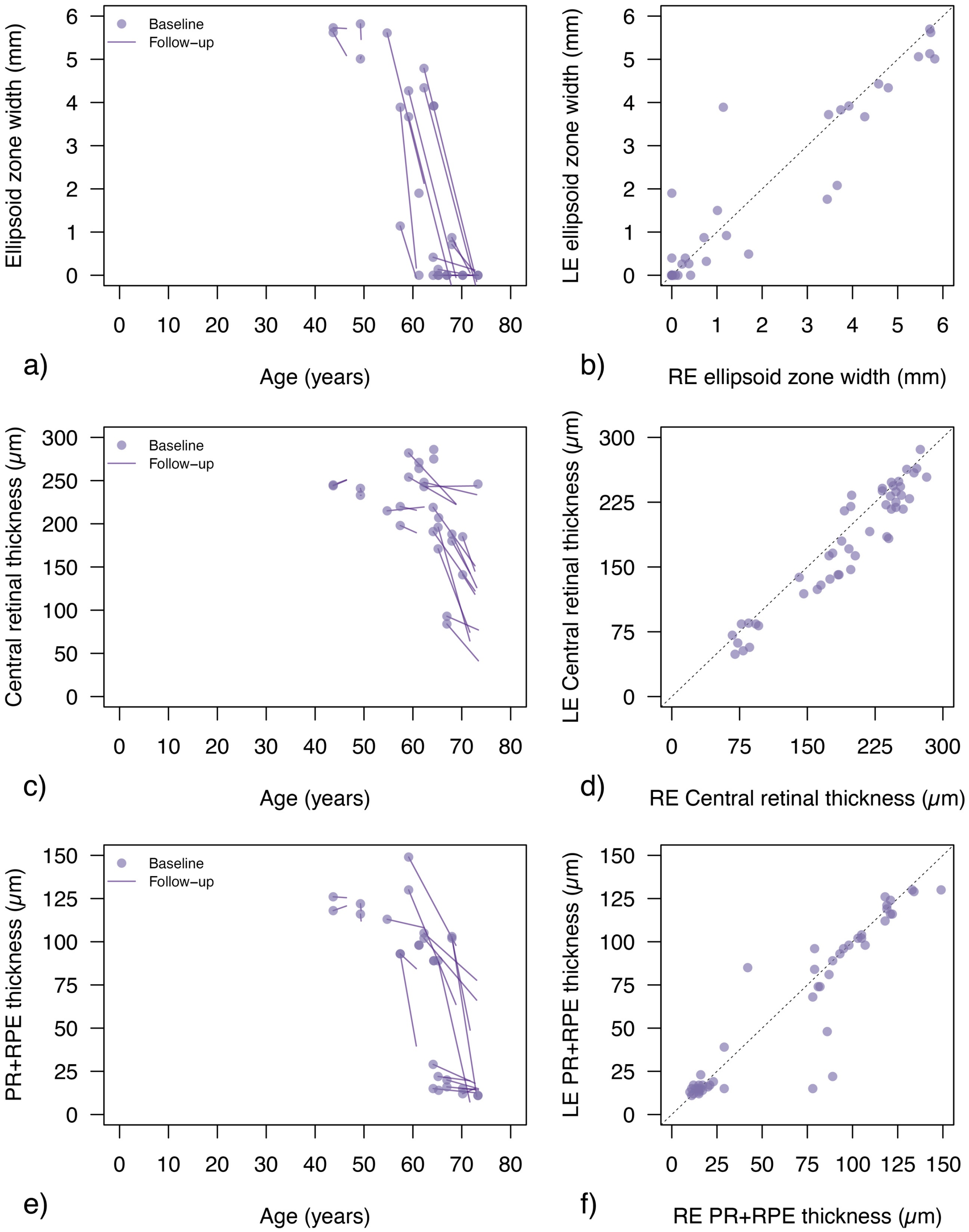

Supplementary Figure 2. Changes in ellipsoid zone (EZ) width, central retinal thickness (CRT) and photoreceptor and retinal pigment epithelium (PR+RPE) thickness by age (a, c, e) and inter-ocular relationship (b, d, f). The plotting is consistent with Figure 3. In (a), points report EZ width at baseline for all eyes. For eyes with follow-up examinations, data at all visits were fitted with linear regression and segments report the corresponding fit for each eye. (b) Inter-ocular relationship for EZ width. A similar representation is reported CRT (c and d), and PR+RPE (e and f).

Supplementary Figure 3

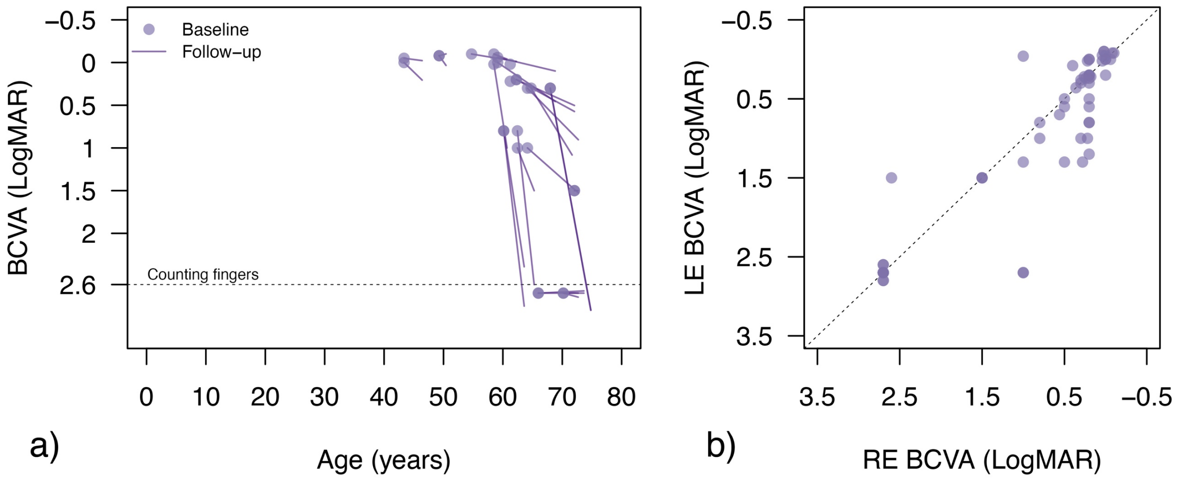

Supplementary Figure 3. Changes in best corrected visual acuity (BCVA) by age (a) and inter-ocular relationship (b). The plotting is consistent with Figure 3, and supplementary Figure 2. In (a), points report BCVA at baseline for all eyes. For eyes with follow-up examinations, data at all visits were fitted with linear regression and segments report the corresponding fit for each eye. The dashed line in reports BCVA level for counting fingers. (b) shows a scatterplot of the inter-ocular relationship for BCVA.
